# Effects of conflict in cognitive control: Evidence from mouse tracking

**DOI:** 10.1177/17470218221078265

**Published:** 2022-02-21

**Authors:** Wenting Ye, Markus F Damian

**Affiliations:** School of Psychological Science, University of Bristol, Bristol, UK

**Keywords:** Cognitive control, conflict adaptation, congruency sequence effect, mouse tracking

## Abstract

It has long been debated whether the “congruency sequence effect (CSE)” in conflict tasks such as Flanker could reflect adaptive control. The current study used “mouse tracking” to tackle the issue in a combination of three conflict tasks (i.e., Flanker, Simon, and Spatial Stroop tasks). Congruency effects from previous and current trials emerged in latencies as well as curvature of movement trajectories in all three tasks. Critically, movement initiation times were affected only by congruency on previous but not on current trials. A further analysis showed that even when initiation time on the previous trials was taken into account, a subtle but highly significant effect of conflict arising from trial *N*–1 on initiation times remained. Although not necessarily implying “conflict adaptation,” i.e., a dynamic up- and downregulation of cognitive control in response to a recent conflict, our finding indicates a specific sensitivity to the presence or absence of recent “conflict” in the cognitive environment.

Cognitive control refers to a set of abilities to direct one’s behaviours in terms of internal goals and context (for overview, see Diamond, 2013). It is commonly believed that cognitive control fractionates into a set of semi-independent components which underlie various aspects of mental skills. For instance, the framework introduced by Miyake et al. (2000) advocates three aspects of executive functions: mental set “shifting,” information “updating,” and “inhibition.” Inhibition is typically investigated via so-called “conflict tasks,” which deliberately induce stimulus-response or stimulus-stimulus incompatibilities. For instance, the seminal Flanker task introduced by Eriksen and Eriksen (1974) requires a speeded response to a centrally presented target stimulus which is surrounded by flanking stimuli which participants are instructed to ignore. A popular version of the Flanker task uses left- or right-pointing arrows as targets and distractors. Response speed and accuracy is detrimentally affected in the case of “incongruent” target/distractors (e.g., <<><<) compared with “congruent” displays (e.g., <<<<<; sometimes “neutral” displays are also used, such as --<--). Other “conflict tasks” include the Simon task, and various versions of Stroop displays such as colour-word and spatial Stroop. The term “conflict” generically refers to an incompatibility which participants have to overcome to successfully perform, and “interference scores” (typically, the difference between incongruent and congruent trials) presumably reflect facets of cognitive control which are relevant for our everyday life abilities to ignore and inhibit irrelevant information (but see Paap et al., 2020, for a critical view).

The notion of “adaptive control” refers to the more specific claim that cognitive control is dynamically adjusted in a time-varying manner, presumably in an effort to reduce the cost of cognitive control only to those circumstances in which it is truly necessary (Shenhav et al., 2013). A yet more specific view of adaptive control is the “conflict adaptation” view (e.g., Botvinick et al., 2001) according to which it is the experience of a *conflict* which induces a transient enhancement of cognitive control. Conflict induces an upregulation of control on following events; by contrast, the absence of a conflict on a given episode downregulates control on subsequent events. The dorsal anterior cingulate cortex (dACC) has been suggested as the brain region which underpins the dynamic regulation of cognitive control in response to the presence or absence of conflict (e.g., Botvinick et al., 2004; Kerns et al., 2004; Shenhav et al., 2013; Sheth et al., 2012). According to this view, conflict is the critical signal which drives dynamic adaptive control (perhaps via eliciting a negative affective reaction; see Dignath et al., 2020).

A key empirical observation which aligns with the notion of “conflict adaptation” is that performance in “conflict tasks” such as Flanker is affected not only by (in-)congruency on a given trial *N*, but also by whether the previous trial (*N*–1) is congruent or incongruent. Specifically, if trial *N*–1 is congruent, then congruency effects on trial *N* are substantial; if trial *N*–1 is incongruent, then congruency effects on trial *N* are much reduced. This sequential pattern is known as the “Gratton effect” (Gratton et al., 1992) or the “congruency sequence effect (CSE),” and it provides *prima facie* strong evidence supporting dynamic and transient adaptation to the presence or absence of conflict. However, the inference that the CSE reflects dynamic adaptation to conflict has been repeatedly challenged. For instance, a problem first highlighted by Mayr et al. (2003) is that the CSE only appears when the responses on trial *N*–1 and on *N* repeat; when responses switch, congruency effects on trial *N* are unaffected by congruency on trial *N*–1 (however, see for instance results from the Simon task reported in Erb & Marcovitch, 2019, where this does not seem to be the case). This led them to postulate that CSEs do not support the “conflict adaptation” view, as originally envisaged by Botvinick et al. (2001), but that instead mind and brain dynamically acquire connections between stimuli and responses. This principle is for instance embedded in a theory advocated by Hommel (2004; Hommel et al., 2004) according to which stimulus and response features are dynamically integrated into “event files.” For example, if the current stimulus and response fully repeat the previous ones (e.g., trial *N*–1: <<<<<, trial *N*: <<<<<), performance can be enhanced because a stimulus-response (S-R) pair has been formed in previous trials and there is no need for formation of a new S-R pair in current trials. However, if there is a partial repetition (e.g., responses repeat but not stimuli: trial *N*–1: <<<<<, trial *N*: >><>>), performance can be impaired because the previous stimulus-response (S-R) pair will interfere with the formation of the current S-R pair when two different stimuli need to be paired with the same response. This line of thinking dispenses with conflict monitoring altogether, but it is also possible that both feature binding and genuine adaptive control contribute to performance, as for instance in the model advocated by Verguts and Notebaert (2009).

Empirical attempts to dissociate “low-level learning” of stimulus-response regularities from genuine cognitive control have highlighted the need for more complex experimental designs and/or tasks than those found in simple “conflict tasks.” For instance, the use of four-alternative-forced choice tasks might avoid the problems of confounding feature association with conflict adaptation (e.g., Kunde & Wühr, 2006; Weissman et al., 2015). Alternatively, classic conflict tasks such as Stroop and Simon can be modified to such that “inducer” items/trials (which trigger adaptive control) alternate with “diagnostic” items/trials (used to measure the effects of adaptive control; see Schmidt & Weissman, 2014, for an example). In other studies (Hazeltine & Mordkoff, 2014; Jacoby et al., 2003; Schmidt, 2013), an attempt has been made to distinguish “item-specific” from “list-wide” proportion congruency effects (the tendency of congruency effect to be reduced in blocks of more- relative to less-frequent incongruent trials). Overall, extant results underscore the challenges of evaluating conflict adaptation in behavioural experiments because latencies typically conflate effects which arise from S-R combinations on a given trial, with those which arise from previous trials (see Braem et al., 2019, and Schmidt, 2019, for recent comprehensive overviews).

An alternative to the use of ever more complex experimental designs is to study cognitive control with methods other than those featuring simple latencies obtained from key press responses, such as electrophysiology (e.g., Scherbaum et al., 2011). Over the last few years, various methods have been developed in which participants provide responses not via key presses but instead via “dynamic” movements carried out via a reaching response to a target, or via responses made on digital tablets or via computer mouse (see Wirth et al., 2020, for a recent overview). Compared with simple key press experiments, these methods offer a much richer, and potentially more informative, picture of the decision-making process as it unfolds. Erb and colleagues (Erb & Marcovitch, 2018, 2019; Erb et al., 2016, 2020) recently reported evidence from various conflict tasks (Stroop, Flanker, Simon) and different age groups (children vs. young adults vs. elderly) using “reach tracking.” In this method, participants carry out responses to congruency displays by reaching to response locations on a digital display while their hand movements are measured by an electromagnetic position and orientation recording system. Variables of interest are not only the latency with which a reaching response arrives at its target, but also its initiation time (measured relative to the onset of the target display), response latency (RT, the time interval between onset of the target display, and clicking on the response field), as well as (and particularly so) the curvature of the movement in space (measured as maximum deviation or MAD, the largest perpendicular deviation between the actual and the idealised trajectories, measured in cm).

Using a Stroop and a Simon task, Erb et al. (2016) reported a dissociation in patterns between initiation and curvature on the basis of which they argued that the classic CSE results from a combination of two distinct processes, a “threshold adjustment process” and a “controlled selection process.” A “response threshold” is raised on an incongruent trial in response to a detection of conflict, and this carries over to the subsequent trial (if the subsequent trial was also incongruent, the threshold would be raised further). As a result of the temporarily raised threshold, motor output is temporarily withheld in response to conflicts from previous and current trials. In their reach tracking study, the hypothesised threshold adjustment process was captured by initiation times, via additive effects of congruency as well as of congruency from the previous trial. In addition, a “controlled selection process” was postulated to resolve conflict by increasing activation of the appropriate S-R bindings while competing responses are co-activated. In their reach tracking study, this mechanism was captured by curvature of reaching responses. Effects of congruency and congruency *N*–1 appeared in curvature, but critically these interacted in the typical fashion of CSEs (effects of congruency were reduced when a previous trial was incongruent) because readily formed S-R bindings might interfere with the current one if they are partially repeated. More specifically, curvatures were smaller on congruent trials, larger on incongruent trials not featuring S-R binding conflict, and largest on incongruent trials featuring S-R binding conflict. The authors argued that in key press experiments, the two cognitive mechanisms of threshold adjustment and selection/monitoring are typically conflated, but that use of a “dynamic” method such as reach tracking allows them to be potentially dissociated.

## The present study

In the study reported below, we tackled the issue of adaptive control via a technique which is related to the “reach tracking” method used by Erb and colleagues. In “mouse tracking” studies, responses are carried out via dynamic movements of the computer mouse (see Freeman et al., 2011, and Schoemann et al., 2021, for overviews). Typically, participants click on a “Start” button at the bottom of a computer screen to initiate a trial, and following presentation of a stimulus, participants move the cursor to one of two response buttons located in the upper left and right edges of the screen. As in “reach tracking,” initiation and response latencies, measures of curvature, plus a plethora of additional characteristics of the responses, can be analysed (see Wirth et al., 2020, for comprehensive overview). Parallels between the two methods are that S-R compatibility such as Flanker effects emerge in curvatures on incongruent trials, via an attraction of trajectories towards the incorrect response field.

One important difference between the two techniques is that in reach tracking, participants tend to initiate movements relatively later relative to stimulus onset than they typically are in mouse tracking studies. In their reach tracking studies, Erb and colleagues reported initiation times of approximately 500 ms post stimulus onset, and these exhibited signs of compatibility/conflict (they were affected by the experimental congruency manipulation). The presence of compatibility or conflict effects in initiation times suggests that participants did not begin a reaching movement until they processed the target display to an important degree. By contrast, in mouse tracking studies, initiation times are typically faster (in our own studies, between 150 and 300 ms) and so it is less likely that a decision has been completed at the time that a response is initiated.^1^ This was explicitly shown in Experiment 1 reported by Wirth et al. (2020) in which in a Simon task, responses made with a computer mouse were initiated quickly (average of 174 ms) but initiation times were not affected by congruency (by contrast, when responses were made on a touchscreen of a tablet computer, initiation times were much slower, with an average of 380 ms, and a congruency effect emerged). The finding concerning responses carried out with a computer mouse converges with numerous mouse tracking data sets collected in our own laboratory and using a range of “conflict” tasks: we generally found fast initiation times which were unaffected by congruency manipulations on a given trial. For instance, Ye and Damian (2022) conducted a colour-shape task switching experiment with mouse tracking and found that congruency effects on a given trial (in task switching: S-R mappings from the task which is not cued on a given trial interfering with responses on the cued task) affected response latencies and curvatures, but not initiation times. By contrast, classic “task switch costs,” i.e., the cost of having just switched from a different to the current task, appeared in initiation times as well as latencies and curvatures.

This general pattern means that in mouse tracking studies, participants tend to initiate a movement before they have fully completed a decision (perhaps for that reason, many highly cited mouse tracking studies such as Freeman & Ambady, 2009, do not even report movement initiation times, presumably because they are not deemed informative). At the same time, initiation times are potentially sensitive to carry-over effects from previous trials, as evidenced by the task switch costs in initiation times reported in Ye and Damian. This is the central aspect of the experiment reported here. It should be noted that tasks with dynamic responses such as mouse tracking and reaching can be set up in various ways, with an important distinction the one between a “static” starting procedure in which a stimulus appears once participants have initiated a trial and participants choose when to initiate a response movement, and a “dynamic” procedure in which the stimulus only appears once participant have initiated their movement (for analysis, see Scherbaum & Kieslich, 2018; Schoemann et al., 2019; see also Scherbaum & Dshemuchadse, 2020). Our argument above regarding the potential sensitivity of movement initiation times in mouse tracking experiments to carry-over effects from a previous trial is specific to the “static” starting procedure (initiation times are generally uninformative with a “dynamic” start procedure) and this is also what we used in the experiment reported below.

In our study, we conducted a mouse tracking experiment in which we compared three “classic” conflict tasks: Flanker, Simon, and Spatial Stroop. For all three tasks, we anticipated substantial congruency effects emerging in response latencies and measures of curvature (because participants are able to reverse movements to the erroneous response field until relatively late in the process, errors are generally rare in mouse tracking experiments). Our critical prediction was that movement initiation times would be unaffected by the congruency manipulation. If so, this would allow us to isolate processing arising as carry-over effects from previous trials. Specifically, we explored whether initiation times might be affected by the presence or absence of incongruency/conflict on the preceding trials, with initiation times slowed down following incongruency/conflict. Such a finding would presumably reflect the action of a dynamic control system which raises or lowers a “response threshold” dependent on the presence or absence of a recent conflict. Importantly, explanations based on dynamically formed stimulus-response associations, such as feature integration (see above), rather than conflict, would be unlikely to be able to account for these findings. This is because the expected null finding regarding an effect of congruency from the current trial on initiation times implies that the current stimulus display has not been fully processed when a movement begins.

We used three different tasks (Flanker, Simon, and Spatial Stroop) to explore whether signatures of adaptive control might differ across various conflict tasks (Braem et al., 2019; Kornblum et al., 1990). All participants carried out all three tasks.

## Method

### Participants

A total of 65 participants (*M*_age_ = 21.08 *SD* = 5.65) were recruited from the student population at University of Bristol. All participants signed on the consent form and agreed to provide their data for further analysis. Participants confirmed that they had normal or corrected-to-normal vision, were not colour-blind, and that they could comfortably use a computer mouse with the right hand (however, we did not elicit information regarding handedness). Ethical approval for this study was granted by the Faculty of Science Research Ethics Committee at University of Bristol (no. 75221).^2^

### Materials, design, and procedure

Flanker, Simon, and Spatial Stroop tasks were adapted so that participants provided their responses via mouse movements rather than key presses. The order of the three tasks was counterbalanced across participants using a Latin square design. Within each task, trials were randomised.

Participants were seated approximately 60 cm from the computer screen and initiated every trial by clicking on a grey “Start” rectangle at the bottom centre of the screen, and the target display appeared immediately following the click. Note that this experimental setup implements a simple “static” starting procedure (see Introduction). They were instructed to respond to the target display by clicking on either top left or top right response area. Participants were also instructed to provide a response as quickly and accurately as they could. The target display would disappear with a response or after 2,000 ms. In each task, there were in total 144 trials, including 2 blocks of 18 practice trials and 3 experimental blocks of 36 test trials. Trials were equally likely to be incongruent, congruent, or neutral (for definition, see below). Participants could take a break between blocks. No feedback regarding response speed or accuracy was provided throughout the experiment.

#### Flanker task

Participants were presented with a string of five arrows, and they were instructed to judge the direction in which the middle arrow pointed and to ignore the other four arrows which could either point in the same direction with the target (“congruent,” e.g., →→→→→), in the opposite direction (“incongruent, e.g., ←←→←←), or consisted of lines (“neutral,” e.g., −−→−−). The size of each stimulus was 171 × 8 pixels, with a distance of 6 pixels between adjacent arrows.

#### Simon task

Participants were presented with a blue or a red square of size 132 × 132 pixels, shown either 630 pixels left or right from the centre of the screen, or centrally. Participants were instructed to judge the colour of the square and to ignore the location of the square: for red square, the response was “left,” and for a blue square it was “right.” Stimulus location coinciding with the response side resulted in a “congruent” trial; stimulus location and response side differing resulted in an “incongruent” trial, and a centrally presented stimulus resulted in a “neutral” trial. In the initial practice block, the words “red” and “blue” were displayed in the left and right response areas, respectively, so that participants could memorise the association between colours and response areas. In the experimental blocks, these cues were not displayed.^3^

#### Spatial Stroop task

Participants were presented with a single arrow (249 × 58 pixels) presented either 742 pixels left or right from the centre of the screen, or centrally. Participants were instructed to judge the direction of the arrow, regardless of its location on the screen. As in the Simon task, coinciding stimulus location and response side resulted in a “congruent” trial, differing location and response side resulted in an “incongruent” trial, and a centrally presented stimulus resulted in a “neutral” trial.

### Apparatus

The experiment was conducted on a PC using the software called *MouseTracker* (Freeman & Ambady, 2010). Stimuli were presented on a 23-inch monitor with 1920 × 1080 screen resolution. The size of the “start” rectangle was 192 × 72 pixels and the size of each response area was 288 × 144 pixels. *MouseTracker* recorded the trajectory of each computer mouse movement with raw *x* and *y* coordinates every 16 ms. Cursor speed, one of the experimental parameters in *MouseTracker*, was set to a value of 12 (with 1 being the slowest and 20 the fastest possible setting).

## Results

**Figure 1.**
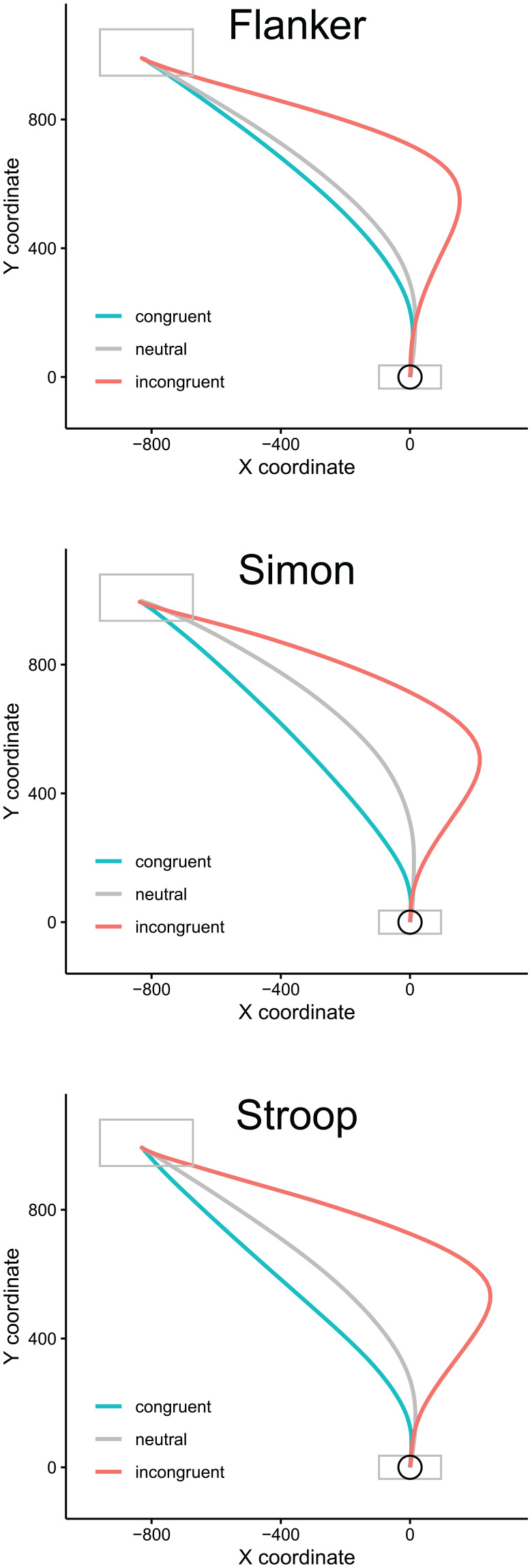
Time-normalised average trajectories, dependent on task (Flanker, Simon, Stroop) and congruency (congruent, neutral, incongruent). Rightward trajectories were flipped to appear as pointing towards the left response. Grey boxes indicate starting and response areas displayed on the computer screen; the circle indicates the virtual starting region relative to which initiation times were computed (see “Results”).

Data were processed in R (R Core team, 2020) using the packages *mousetrap* (Kieslich et al., 2019) and *afex* (Singmann et al., 2016). For every response on each trial, its accuracy, initiation time, RT, and MAD were computed. We observed that mouse clicks on the “start” region are often associated with miniscule movements which are then erroneously recorded by MouseTracker as very “early” (close to or at zero milliseconds) initiation times. For this reason, we defined initiation time as the first time sample relative to target onset in which the mouse cursor left a starting region. As described under “Methods,” the starting region presented on the computer screen consisted of a rectangular box at the centre bottom of the screen. According to Wirth et al. (2020), a rectangular “start” region might be not optimal because initiation times could be confounded with starting angle of a movement and hence a circular starting region would be preferable. We followed their recommendation and computed initiation times as the first time sample at which movements left a virtual circular region with a diameter equal to the height of the response box (see Figure 1). However, all results below were statistically equivalent when initiation times were calculated relative to the actual response box.

We excluded data from the first trial of each experimental block (2.8%), given that these were not preceded by another trial. Overall error rate was very low (0.5%) and hence errors were not further analysed. Data on trials with incorrect responses were excluded from analysis of all other measures. We also excluded data from trials following errors (0.3%) to control for potential post-error adjustments (e.g., Danielmeier & Ullsperger, 2011). Finally, we excluded trials with response latencies faster than 250 ms or slower than 2,000 ms (0.4%) and initiation times slower than 500 ms (0.8%). In total, therefore, 4.8% of data were excluded from the analyses below. Raw data and R analysis files are available on the Open Science Framework.

Figure 1 displays time-normalised average trajectories for each task and each of the three levels of congruency. Rightward trajectories were flipped to appear as pointing towards the left response. The results show the pattern expected from earlier mouse tracking studies such as Damian et al. (2018), with considerably more “curved” responses in the incongruent than in the congruent and neutral conditions.

A descriptive summary for the three dependent measures of initiation times, response latencies, and curvature (MAD) is provided in the online Supplementary Material A. Table 1 provides the results of a four-way analysis of variance (ANOVA) conducted on initiation times, with task, congruency, congruency N–1, and response repeat; for the latter variable, trials were re-coded regarding whether a response was the same (“response repeat”) or different (“response switch”) as the response on the preceding trial. Table 2 provides parallel results for response latencies and MAD. Each ANOVA involves a total of 15 statistical tests (4 main effects and 11 interactions). To control the Type I error rate, Benjamini-Hochberg corrections were applied to the *p* values (e.g., Cramer et al., 2016).

**Table 1.** Analysis of variance performed on initiation times, with task (Flanker, Simon, Stroop), congruency (congruent, neutral, incongruent), congruency on previous trial (*N*–1; congruent, neutral, incongruent), and response repeat (switch vs. repeat).

Effect	df_1_	df_2_	Initiation times			
			mean squared errors (MSE)	*F*	$\eta_{p}^{2}$	*p*
Task	2	128	**18,421**	**75.9**	**.54**	**<.001**
Congruency	2	128	919	1.2	.02	.63
Congruency *N*–1	2	128	**1,110**	**13.6**	**.18**	**<.001**
Response repeat	1	64	1,152	6.1	.09	.08
Task × congruency	4	256	858	0.1	<.01	.97
Task × congruency *N*–1	4	256	1,029	1.3	.02	.63
Congruency × congruency *N*–1	4	256	1,009	0.3	.00	.96
Task × response repeat	2	128	853	2.7	.04	.28
Congruency × response repeat	2	128	1,223	1.1	.02	.64
Congruency *N*–1 × response repeat	2	128	978	0.1	<.01	.96
Task × congruency × congruency *N*–1	8	512	1,009	1.6	.03	.34
Task × congruency × response repeat	4	256	1,089	0.6	.01	.96
Task × congruency *N*–1 × response repeat	4	256	1,194	0.3	<.01	.96
Congruency × congruency *N*–1 × response repeat	4	256	1,359	0.6	.01	.96
Task × congruency × congruency *N*–1 × response repeat	8	512	944	0.5	.01	.96

Significant effects are bolded.

**Table 2.** Analysis of variance performed on response latencies and trajectory maximum deviation, with task (Flanker, Simon, Stroop), congruency (congruent, neutral, incongruent), congruency on previous trial (*N*–1; congruent, neutral, incongruent), and response repeat (switch vs. repeat).

Effect	df_1_	df_2_	Response latencies				Maximum deviation			
			MSE	*F*	$\eta_{p}^{2}$	*p*	MSE	*F*	$\eta_{p}^{2}$	*p*
Task	2	128	**36,067**	**176.60**	**.73**	**<.001**	55.93	1.46	.02	.30
Congruency	2	128	**8,065**	**467.51**	**.88**	**<.001**	**52.98**	**898.54**	**.93**	**<.001**
Congruency *N*–1	2	128	**3,594**	**4.97**	**.07**	**.02**	**14.76**	**16.75**	**.21**	**<.001**
Response repeat	1	64	12,563	1.14	.02	.31	128.19	0.11	.0	.77
Task × congruency	4	256	**5,915**	**12.85**	**.17**	**<.001**	**27.38**	**21.44**	**.25**	**<.001**
Task × congruency *N*–1	4	256	4,891	2.14	.03	.13	20.12	0.96	.02	.50
Congruency × congruency *N*–1	4	256	**4,986**	**11.59**	**.15**	**<.001**	**17.80**	**14.57**	**.19**	**<.001**
Task × response repeat	2	128	8,014	1.34	.02	.31	**46.60**	**9.69**	**.13**	**<.001**
Congruency × response repeat	2	128	**4,944**	**14.27**	**.18**	**<.001**	**19.43**	**6.01**	**.09**	**.01**
Congruency *N*–1 × response repeat	2	128	**5,001**	**9.27**	**.13**	**<.001**	**19.06**	**20.84**	**.25**	**<.001**
Task × congruency × congruency *N*–1	8	512	4,973	0.81	.01	.60	19.09	1.54	.02	.20
Task × congruency × response repeat	4	256	4,966	1.89	.03	.16	16.25	0.45	.01	.77
Task × congruency *N*–1 × response repeat	4	256	4,703	1.55	.02	.23	**14.15**	**16.38**	**.20**	**<.001**
Congruency × congruency *N*–1 × response repeat	4	256	**4,549**	**5.96**	**.09**	**<.001**	**15.03**	**6.16**	**.09**	**<.001**
Task × congruency × congruency *N*–1 × response repeat	8	512	4,774	1.63	.03	.16	16.06	1.52	.02	.20

Significant effects are bolded.

### Initiation times

Results showed a significant main effect of task, with initiation times differing between the three tasks (Flanker: 242 ms; Simon: 190 ms; Stroop: 176 ms). Tukey-corrected post hoc comparisons showed that initiation times in all three tasks differed significantly from one another; *t*s ⩾ 2.51, *p*s ⩽ .035. The results also showed a significant main effect of congruency *N*–1. Tukey-corrected comparisons showed that the congruent and incongruent condition differed significantly from one another; Δ = 5 ms, *t*(128) = 3.58, *d* = .48, *p* = .001. The neutral and the incongruent condition also differed significantly, Δ = 7 ms, *t*(128) = 5.07, *d* = .54, *p* < .001, but the neutral and the congruent condition did not, Δ = 2 ms, *t*(128) = 1.49, *d* = .09, *p* = .300. No other main effects or interactions were significant. Of particular importance, congruency was not significant, nor was it involved in any higher order interactions.

We additionally conducted a Bayesian analysis using the package *BayesFactor* (Morey & Rouder, 2018) with the function *anovaBF()*. Of critical interest were the two variables congruency and congruency *N*–1. The Bayes factor for congruency was *BF*_10_ = 0.006, representing “extreme” evidence for the null hypothesis that initiation times were not affected by congruency. *BF*_10_ for the variable congruency *N*–1 was 23.2, representing “strong” evidence that this variable did affect initiation times.

### Response latencies and curvature

These showed a considerably more complex pattern than the one obtained for initiation times. Specifically, Table 2 shows a highly significant interaction between congruency and congruency *N*–1 (the classic “congruency sequence” or “Gratton” effect), as well as the three-way interaction between congruency, congruency *N*–1, and response repeat first highlighted by Mayr et al. (2003). Further worth highlighting is an interaction between task and congruency in latencies and curvature (the latter is visible in Figure 1 in terms of somewhat different conditional average trajectories in the three tasks), which suggests that the three tasks exhibited subtle differences in terms of how congruency affected performance. Crucially, neither RTs nor MAD showed a four-way interaction between congruency, congruency *N*–1, response repeat, and task. This implies that the important three-way interaction between congruency, congruency *N*–1, and response repeat does not differ substantially across the three tasks.

**Figure 2.**
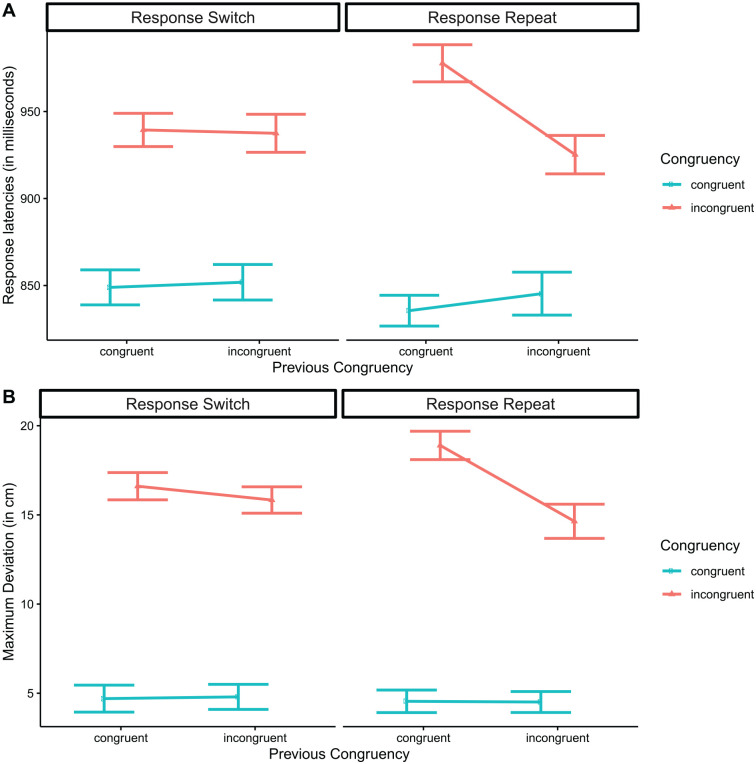
Three-way interaction between congruency, congruency *N*–1, and response repeat. Response latencies (a) and maximum deviation (b). The “neutral” condition has been omitted for both congruency and congruency *N*–1. Error bars in inset panels reflect 95% confidence intervals.

Figure 2 shows the critical three-way interaction, separately for RTs (Figure 2a) and MAD (Figure 2b). For ease of exposition, in this figure we omitted the “neutral” condition for both congruency and congruency *N*–1. The characteristic pattern, with an interaction between congruency and congruency *N*–1 prominent for “response repeat” responses but weaker or perhaps absent in “response switch” responses (e.g., Erb & Marcovitch, 2018; Mayr et al., 2003), was also found here.

The three-way interaction in response latencies was followed up with simple effects of congruency and congruency *N*–1, conducted separately for “response switch” and “response repeat” trials. For “response switch,” we found main effect of congruency, *F*(2, 128) = 218.02, *p* < .001, no effect of congruency *N*–1, *F* = 1.15, and no significant interaction, *F*(4, 256) = 2.07, *p* = .127. For “response repeat” trials, we found effects of congruency, *F*(2, 128) = 368.75, *p* < .001, of congruency *N*–1, *F*(2, 128) = 7.09, *p* = .001, and an interaction, *F*(4, 256) = 20.42, *p* < .001. Follow-up analysis of the interaction showed that “congruent” trials were significantly affected by congruency *N*–1, *t*(380) = 2.78, *d* = .20, *p* = .016, and so were “incongruent” trials, *t*(380) = 7.75, *d* = .73, *p* < .001.

To further explore this pattern, we computed “conflict adaptation effects,” as described in Nieuwenhuis et al. (2006, p. 1,261) via the equation:

$$\begin{array}{l} \mathrm{Conflict}\;\mathrm{adaptation}\;\\ \;\;\;\;\;\;\mathrm{effect}\;=\;\left({\mathrm{RT}}_{\mathrm{CI}}-{\;\mathrm{RT}}_{\mathrm{CC}}\right)\;-\;\;\left({\mathrm{RT}}_{\mathrm{II}}-{\;\mathrm{RT}}_{\mathrm{IC}}\right) \end{array}$$

where the first subscript index indicates congruency on trial *N*–1, and the second index indicates congruency on trial *N*. For instance, RT_CI_ indicates the response latency on an incongruent trial preceded by a congruent trial. The prediction from Nieuwenhuis et al. (2006) and others is that the conflict adaptation effect should be close to zero for “response switch” responses, but it should be substantial for “response repeat” responses (note that for this analysis, the “neutral” condition was omitted). Our calculation showed a conflict adaptation score of 1 ms for the “response switch” condition, *t*(64) = 0.11, *d* = .01, *p* = .915, and of 77 ms for the “response repeat” condition, *t*(64) = 6.26, *d* = .78, *p* < .001. Equivalent conflict adaptation effect scores computed for MAD rather than RTs showed a similar pattern, with a very low score (0.7 cm) for “response switch” responses, *t*(64) = 1.35, *d* = .17, *p* = .181, but a much higher score (4.3 cm) for “response repeat” responses, *t*(64) = 7.84, *d* = .97, *p* < .001.

Overall, we replicated the critical pattern reported previously by, e.g., Erb and Marcovitch (2018), Mayr et al. (2003), and Nieuwenhuis et al. (2006): the CSE pattern was present when responses on trial *N*–1 and *N* were “same,” but not when they were “different.”

#### Interim summary

In all three tasks, response latencies as well as response movement characteristics showed the expected complex pattern previously reported in the literature. Specifically, a “congruency sequence” (or “Gratton”) effect emerged in the key interaction between congruency and congruency *N*–1, originally taken as an indicator of “conflict adaptation” (Botvinick et al., 2001). We further found the signature three-way interaction between congruency, congruency *N*–1, and response repetition, previously highlighted to be problematic for the classic “conflict adaptation” account (e.g., Hommel, 2004; Mayr et al., 2003). Effects in latencies and in response curvatures were largely in agreement. By contrast, response initiation times presented with a considerably simpler pattern, with a small but highly reliable effect of congruency *N*–1, and a main effect of task, which will be examined in the Discussion. Critically, congruency did not affect initiation times. As expected from previous studies (see Introduction), responses were initiated so early (<300 ms) that stimulus-driven variables such as congruency did not yet have an impact.^4^

The effect of congruency from the previous trial onto initiation times on a current trial may be taken as reflecting cognitive sensitivity to “conflict.” By making initiation times the main dependent variable of interest, we untangled effects arising from a current trial (congruency) from those arising from the previous trial (congruency *N*–1). And the finding that congruency *N*–1 exerts a subtle but highly reliable effect on initiation times is generally in line with the notion of “conflict adaptation” in the sense originally postulated by Botvinick et al. (2001) according to which the cognitive control system dynamically responds to the presence or absence of a conflict by up- or downregulating control. Having said that, if our interpretation of the results is correct, they do not reflect “conflict adaptation” per se, which would require the demonstration of a modification of cognitive control on a given trial based on presence/absence of conflict on a previous trial such as in the original interpretation of the CSE. Rather, the dynamic adjustment of the response threshold which is visible in our results forms a central component of models of cognitive control which attribute importance to conflict detection and monitoring (e.g., Botvinick et al., 2001; Erb & Marcovitch, 2018).

There is, however, an alternative explanation for our findings which needs to be considered. According to Schmidt and Weissman (2016), human behaviour exhibits “temporal learning” in the sense that participants will learn not only which responses to make, but also *when* to make them. Sequential (or “autocorrelational”) effects in experimental studies are well documented (e.g., Kinoshita et al., 2008), and the general finding is that slow responses on trial *N*–1 are followed by slow responses on trial *N*, and the same for fast responses. In this sense, a good portion of the variance of behaviour is predictable from the responses which just previously occurred. According to Schmidt and Weissman, a CSE in key press experiments gives the appearance of resulting from dynamic adjustment of cognitive control, but it could instead be produced by temporal learning in the following way. On a given trial, activation of response alternatives builds up across time, and a response is determined by a “response threshold” which needs to be crossed. This response threshold is dynamically adjusted on each trial, based on response time on the preceding trial(s). Following a “congruent” *N*–1 trial, the response threshold is dropped earlier than following an “incongruent” *N*–1 trial. Following a “congruent” *N*–1 trial, on a “congruent” trial *N* response activation builds up quickly and hence a response benefits from the reduced threshold which occurs at an early point in time. Activation on an “incongruent” trial *N* will accrue more slowly and hence may not benefit from the reduced threshold. If so, this would result in a marked response time difference between congruent and incongruent trials *N*, when preceded by a congruent trial. By contrast, following an “incongruent” trial *N*–1, the response threshold is also dropped but at a later point in time compared with a “congruent” trial *N*–1. Here, an “incongruent” trial *N* might benefit from the dropped threshold because activation builds up with additional time, but a “congruent” trial *N* might not because the response has already been made when the response threshold drops. This would result in a small congruency effect. Hence, according to this account, the CSE effect in key press latencies arises not from conflict adaptation but rather from the sensitivity of cognitive processing to temporal aspects of a context.

If this account is correct, then the presence of conflict on trial *N*–1 is confounded with the fact that incongruency slows down responses on that trial. Schmidt and Weissman (2016) reanalysed data from a prime-probe task and showed via an analysis conducted on raw data that once response time on trial *N*–1 was included as a predictor, then congruency *N*–1 no longer significantly predicted latencies on trial *N*. These findings were taken as evidence against the notion of “conflict adaptation.” In a computational simulation, Schmidt and Weissman demonstrated that a model with a dynamic response threshold which is adjusted based on “episodic nodes” which store information about RTs on previous trials can indeed simulate the empirical pattern. Critically, the model requires no “conflict monitoring” structure to accomplish this goal, and the authors concluded that “it is important for researchers to entertain the possibility that other mechanisms besides conflict adaptation can explain the CSE” (p. 605).

It is worth considering how this idea might transfer to studies such as our own which implement a “dynamic” response such as those made via computer mouse movements. In key press experiments such as those analysed and modelled by Schmidt and Weissman (2016), a response threshold presumably determines the point in time at which enough activation has accrued from the stimulus display to determine the response. This makes intuitive sense and is a central component of formal models of response times such as the “diffusion model” by Ratcliff and colleagues (see Ratcliff et al., 2016, for overview). By contrast, in experimental studies with “dynamic” responses, it is possible that a movement is initiated before a decision has been completed. Indeed, our results reported above which show that initiation times are not affected by congruency on trial *N* suggest this to be the case. Hence, contrary to key press experiments, in a mouse tracking study the “response threshold” which determines the point at which participants begin moving the computer mouse is not primarily determined by sufficient activation about the correct response having been accrued. Nonetheless, our results show that initiation times are sensitive to congruency on the previous trial, which plausibly reflects dynamic adjustment of the response threshold as suggested by Schmidt and Weissman.

Hence the question arises: could our central finding of an effect of congruency on trial *N*–1 on initiation times be unrelated to the presence or absence of a *conflict* on trial *N*–1, but purely arise from “temporal learning” in the sense suggested by Schmidt and Weissman (2016)? According to this account, it is irrelevant whether or not a conflict occurred on trial *N*–1; rather, the speed of initiation times on trial *N*–1 will predict much of the variability of initiation times on trial *N*, and once this variable has been included in the analysis as a predictor, then the effect of congruency *N*–1 will disappear.

To put this prediction to the test, we conducted a further analysis of our results which was based on raw initiation times (rather than conditional averages). We applied a linear mixed effects model analysis in which initiation times on a given trial were predicted by initiation times on trial *N*–1 (we also included task because this variable was significant in the ANOVA reported in Table 1). Critically, we re-coded “congruency” on trial *N*–1 in terms of “conflict” (incongruent condition) and “no conflict” (congruent and neutral condition). To meet the distributional assumption of a linear mixed model, the dependent variable has to exhibit an approximately normal distribution. As described above, initiation times larger than 500 ms (0.8% of all data points) had already been trimmed prior to the analysis. Visual inspection of the remaining movement initiation times (see Figure 4 in the online Supplementary Material B for a histogram) showed a reasonably symmetric distribution with only a moderate amount of right skewness (0.35; by comparison, response latencies showed a much larger degree of skewness, 0.96). Various transformations which we conducted on initiation times in an attempt to further approximate a normal distribution (inversion; log transformation) lead to a worse outcome than with the non-transformed data. Therefore, the results reported below come from an analysis of non-transformed initiation times. However, the trimming of initiation times larger than 375 ms (5.5%) lead to an almost perfectly symmetric distribution of the remaining data points (skewness of 0.05; see Figure 4 in the online Supplementary Material B) and statistics showed equivalent results to the untrimmed version reported below.

A linear mixed-effects model was constructed, with initiation time on trial *N* as the dependent variable, and the variables “task” and “conflict on trial *N*–1” as fixed effects. Critically, initiation time on trial *N*–1 was included as a fixed effect. Participants were entered as the random effect. Initiation times *N*–1 were centred on the mean to avoid correlation with the intercept (Baayen, 2008, p. 254). We used the *mixed()* function from package *afex* with the Satterthwaite method used to calculate *p* values. The results showed a main effect of task, *F*(2, 19971) = 609.12, *p* < .001, of initiation time on trial *N*–1, *F*(1, 20002) = 1,513.93, *p* < .001, and critically of conflict on trial *N*–1, *F*(1, 19959) = 33.12, *p* < .001. The only interaction which reached significance was between task and initiation time on trial *N*–1, *F*(2, 19969) = 30.97, *p* < .001; none of the other interactions were significant, *F*s ⩽ 2.50, *p*s ⩾ .083. The estimate for the effect of conflict on trial *N*–1 from the best fitting model was 6 ms.^5^

We further quantified the contribution made by the factor conflict on trial *N*–1 via Bayesian analysis, using the package *BayesFactor* (Morey & Rouder, 2018) with the function *lmBF()*. The critical comparison derived from the linear mixed effects analysis above was the one between a restricted model which contained the variables task and initiation time on *N*–1 as interactive terms, to a full model which additionally included the variable conflict on trial *N*–1. Participants were again included as the random effect. The comparison showed a Bayes Factor of *BF*_10_ > 1,000 in favour of the full relative to the restricted model, yielding “extreme” support for the hypothesis that initiation times on a given trial were affected by conflict on trial *N*–1.

**Figure 3.**
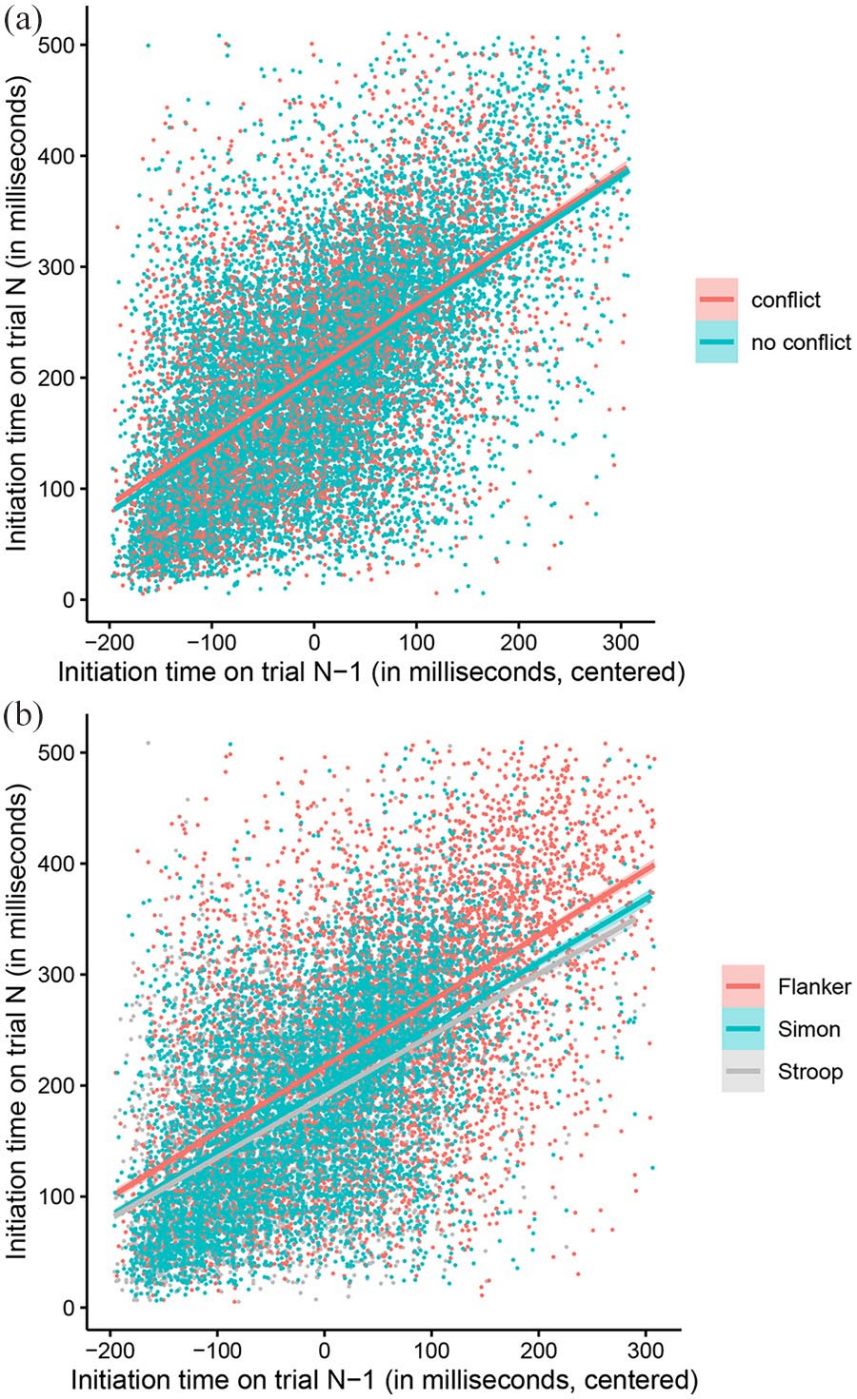
Raw initiation times on trial *N*, dependent on centred initiation time on trial *N*–1. Effect of “conflict” (a) and of “task” (b). Data points are multiples of the sampling rate (16 ms) and have been slightly jittered to avoid overlapping.

Figure 3 shows the relationship between initiation time on trial *N*–1 and on trial *N*; the strong temporal dependency between the two measures is readily apparent (Adjusted *R*^2^ = .37). Figure 3a shows the effect of conflict on trial *N*–1, whereas Figure 3b shows the main effect of task. The coefficients for the relation between initiation times *N*–1 and *N* for the Flanker, Simon, and Stroop task were .59, .57, and .55 respectively, *p*s < .001.

## Discussion

The current study compared three “classic” conflict tasks widely used in the literature (Flanker, Simon, Spatial Stroop) in a paradigm in which participants responded with movements made by the computer mouse (“mouse tracking”). The results replicated previously reported “congruency sequence effects” (CSEs) in RTs and curvatures via an interaction between congruency and congruency *N*–1, and they also showed a characteristic three-way interaction between congruency, congruency *N*–1, and “same” versus “different” responses between trials *N*–1 and *N* (e.g., Mayr et al., 2003). Critically, initiation times revealed a different pattern, such that only task and congruency *N*–1 showed significant effects. As predicted from the observation that in mouse tracking experiments, participants tend to initiate their response movements relatively soon after the beginning of a trial, congruency on a given trial did not yet have an impact on initiation times. This suggests that a decision based on the target display had not yet been made when the response began. However, congruency from the previous trial showed a carry-over effect on initiation times, with slower initiation times after incongruent than congruent trials. This finding does not directly demonstrate “conflict adaptation” in the strict sense originally postulated by Botvinick et al. (2001), i.e., a dynamic up- and downregulation of cognitive control in response to a recent conflict. Nonetheless, initiation times appeared to be sensitive to conflict on previous trials.

We considered an alternative explanation for our findings which does not rely on the notion of conflict adaptation but rather arises from the notion of “temporal learning.” According to this concept, human behaviour generally shows sequential characteristics, such that response times on a given trial are to a large extent predictable from previous responses. If so, then responses (in this case: initiation times) on trial *N* might simply be predictable from responses (initiation times) on the previous trial. We tested this notion via a linear mixed-effects model analysis and found that temporal learning indeed played a role: initiation times on trial *N* were strongly predictable from initiation times on trial *N*–1. There was also a main effect of task and a significant interaction between task and initiation times on *N*–1, implying that the carry-over effect of previous initiation times on current initiation times depends to some extent on the task. Critically, however, the effect of conflict on the previous trial was still highly significant once initiation times on trial *N*–1 had been included as a predictor. Such carry-over effects on initiation times would be difficult to be explained by temporal learning alone. We further note that it is unclear whether the temporal learning account could explain the complex interplay between initiation times and response latencies (i.e., additive effects of congruency *N*–1 and *N* on initiation times; an interactive pattern on latencies) documented in reach tracking experiments such as the one reported in Erb et al. (2016).

The effect of presence/absence of conflict on a previous trial onto initiation times on a current trial indicates cognitive sensitivity to conflict. This observation could be taken as support for the notion of “conflict adaptation” in the sense originally postulated by Botvinick et al. (2001) according to which the cognitive control system dynamically responds to the presence or absence of a conflict by up- or downregulating control. However, in our view, this implies overinterpretation of our findings. The original interpretation of empirically observed CSE effects (e.g., Botvinick et al., 2001; Kerns et al., 2004) was that they reflected a conflict monitoring system which was functionally adaptative. For instance, Kerns et al. characterised conflict adaptation such: “ . . . monitoring of response conflict acts as a signal that engages control processes that are needed to overcome conflict and to perform effectively” (p. 1023). In this sense, “adaptative” implies an improvement in performance, as indicated by reduced conflict scores following recent conflict. By contrast, our own central finding—a sensitivity of movement initiation times to the presence or absence of recent conflict—suggests that conflict is relevant in the cognitive environment (contra, e.g., Schmidt, 2019) but our effect does not warrant being characterised as “adaptive” in a top-down sense. Indeed, Erb and Marcovitch (2018) characterised their “response threshold process” as being conflict-triggered but not involving top-down cognitive control. Our central observation of the current paper might fit such a definition more closely than the notion of “adaptive control” originally taken to account for CSE effects.

The overall pattern of results in our mouse tracking experiment is in agreement with computational models of behaviour that assume continuous information flow and parallel processing between cognitive processing stages, and specifically an “adaptive flow of information” between the final cognitive processing stage and motor response (Calderon et al., 2018; Eriksen & Schultz, 1979; for a review, see Erb et al., 2021). It appears that at the point when individuals initiate a response carried out via the computer mouse, they have not yet accumulated enough evidence for the ultimate decision, as evidenced by the null finding of congruency on initiation times. Initiation of a response is presumably based on the crossing of some sort of “response threshold,” but the use of this term in the current context is potentially confusing. A “response threshold” is a central component of most formal models of decision making. For instance, in the “diffusion model” by Ratcliff and colleagues (Ratcliff et al., 2016), evidence for an alternative accumulates until a critical boundary is reached, and this boundary is evidently flexible, accounting for speed-accuracy trade-offs (e.g., Bogacz et al., 2010). By contrast, in experiments with “dynamic” responses such as reach and mouse tracking, a response can be initiated prior to a commitment to a specific response. In the model advocated by Erb and colleagues outlined in the Introduction (e.g., Erb & Marcovitch, 2018), a response threshold is dynamically adjusted in response to the presence/absence of a cognitive conflict. In this model, on incongruent Flanker trials, the “direct pathway” generates relatively strong activation corresponding to the distractor arrows, and relatively weaker activation corresponding to the central target arrow. A monitoring device registers this conflict, and by temporarily raising the response threshold, halts motor production until the conflict has been resolved in favour of the response to the target. Hence, a conflict could be registered before a decision has been completed. Similarly, in our mouse tracking study, it appears that a response is initiated before decision making has been completed. Hence, although presumably some sort of boundary needs to be crossed before participants start moving the mouse, this threshold appears fundamentally different from the one postulated for key press experiments.

Our findings pose an interesting contrast when compared with those reported by Weissman et al. (2015). These authors utilised a conflict task in which the impact of learning and memory confounds on performance was minimised, and they contrasted a version in which target and distractor (both arrows) were either presented simultaneously or the distractor was given a processing “head start.” A CSE was found only in the sequential version. Interestingly, their third experiment contrasted very brief (33 ms) and quite long (1,000 ms) delays between prime and probe, and showed a CSE of very similar magnitude under both delays. Importantly, with the long delay, mean latencies for congruent and incongruent trials were virtually identical, which was taken to be incompatible with an explanation of the CSE in terms of “temporal learning” (see above). In that sense, ours and their findings agree in their inference that temporal learning cannot account for the full pattern of known results. However, with the long delay they also found a “reverse congruency” effect (a positive congruency effect following congruent trials, and a negative congruency following incongruent trials) which is problematic for explanations based on attentional shift (among them, the classic “conflict adaptation” view) because perfect cognitive control might eliminate a distractor influence, but not reverse its influence. Given that our own findings underscore the potential importance of recent conflict in the cognitive environment, future research will have to determine how the two sets of results can be reconciled.

Behavioural performance in tasks with “dynamic” responses such as mouse tracking is clearly subject to task- and procedure-specific aspects. For instance, Scherbaum and Kieslich (2018) demonstrated important differences in outcome when comparing a “static” and a “dynamic” starting procedure in a mouse tracking study (see Kieslich et al., 2020, for a further exploration of potentially relevant experimental aspects of mouse tracking, and Wirth et al., 2020, for general design recommendations for mouse- and finger-tracking studies). In the specific format of our own mouse tracking study reported here (participants self-initiated each trial by clicking on a “Start” region; the display appeared immediately upon initiation of the trial; no feedback concerning RTs or errors was given, etc.), it is empirically the case that congruency manipulations on a given trial do not affect initiation times, suggesting to us that responses are initiated before evidence regarding the ultimate decision has accumulated. And this observation allowed us to use initiation times as an indicator of conflict on a previous trial. However, the observed insensitivity of initiation times to congruency/conflict on a given trial is clearly not an inherent property of the mouse tracking technique. Hence, it would be inappropriate to conclude based on the current findings that mouse tracking is superior to alternative methods (e.g., reach tracking) in capturing the decision-making process in conflict tasks.

If not the accumulation of sufficient evidence to make a decision, what determined the time at which time a computer mouse response is initiated in our study? Our results (Table 1) showed that initiation times were affected by congruency/conflict on the previous trial, but also by task, with slowest average initiation times in the Flanker task (242 ms) and considerably faster averages in the Simon (190 ms) and Stroop task (172 ms). Hence, initiation of a response appears sensitive to the general task demands and characteristics of a specific context, even if on each given trial, the specific stimulus display has not been processed to an extent that congruency effects would emerge (see the absence of congruency effects on initiation times). In our intuition, participants choose an initiation time which will allow them to complete the response in a single sweeping movement (i.e., they avoid movement disruption). In other words, the general demands of a task set the “criterion” for initiation times, and congruency/conflict on the previous trial can modify it slightly.

In their key-press experiment, Schmidt and Weissman (2016) used response latency on the previous trial, as well as congruency, to predict response latency on the current trial (and concluded that congruency was no longer relevant once latency *N*–1 had been taken into account). By contrast, the main focus in our own study was on initiation times rather than response latencies. One may ask why we used initiation time *N*–1, rather than response latency *N*–1, as a predictor for initiation times on trial *N*. There were two reasons for this choice. First, according to the “temporal learning” view advocated by Schmidt and Weissman, the “rhythm” of a response in a trial is learned and applied to subsequent trials. Hence, it is likely that in addition to the length of a response (i.e., RT), *when* to initiate a response (i.e., initiation times) will also be affected by the previous response. Second, on a given trial *N*, RT is affected by congruency/conflict, and hence RT on trial *N*–1 is equally affected by congruency/conflict on trial *N*–1. This implies that RT *N*–1 is not a good predictor because congruency/conflict and RT are conflated. By contrast, initiation times are generally unaffected by congruency/conflict on a given trial, and so will initiation times *N*–1 be unaffected by congruency/conflict *N*–1. This makes initiation times *N*–1 the ideal predictor to see whether conflict has an independent effect, above and beyond simple temporal dependencies.

In our results, sequential (“autocorrelational”) effects emerged strongly, both in initiation times and in response latencies. Sequential effects of this type are well documented in key-press experiments (e.g., Kinoshita et al., 2008), but we were surprised at the strength of these effects in our mouse tracking methodology. The correlation between initiation time on current and preceding trials was *r* = .61, *p* < .001 (see Figure 3), and the correlation between response time on current and preceding trials was *r* = .41, *p* < .001. At the same time, autocorrelational effects between the two measures were very small: the correlation between initiation time, and response latency on the preceding trial, was *r* = .21, *p* < .001, and the correlation between response latency, and initiation times on the preceding trial, was *r* = .16, *p* < .001. It appears that when a response movement is initiated, and how fast/slow it is completed, reflect different processing characteristics. The strength of the autocorrelational patterns in our data was particularly surprising to us given the fact that in our version of the mouse tracking paradigm, each trial was self-initiated (via participants clicking on the “Start” field) and hence the intertrial interval was variable. The software which we used to collect our data time stamps the beginning of each trial only in full seconds, hence we were able to obtain merely a rough measure of interstimulus and response-stimulus intervals. Our estimates computed from the time stamps for the ISI (interstimulus interval) and RSI (response-stimulus interval) are 2,068 and 1,186 ms. The latter suggests that participants took on average only slightly more than a second to return the cursor from a response field back to the “Start” button and to ready themselves for the next trial. Hence, participants executed trials at a rapid pace which may well be comparable to the one in conventional key press experiments.

Our study employed three different “conflict tasks” (Flanker, Simon, spatial Stroop). In response latencies, we found a main effect of task (Flanker: 967 ms, Simon: 849 ms, Stroop: 832 ms) as well as an interaction between task and congruency (incongruent minus congruent condition: Flanker: 121 ms, Simon: 97 ms, Stroop: 98 ms). These effects are generally in line with previous findings from key press or mouse tracking studies (e.g., Damian et al., 2018, and Zhou & Krott, 2018, also found that RTs were slowest in the Flanker task, relative to Simon and Stroop tasks). An interesting and theoretically relevant additional findings is that we obtained the characteristic interaction between congruency and congruency *N*–1 (the CSE or “Gratton effect”) as well as a three-way interaction between congruency, congruency *N*–1, and response repeat (highlighted first by Mayr et al., 2003); however, there was no interaction between congruency, congruency *N*–1, and task, nor a four-way interaction between congruency, congruency *N*–1, response repeat, and task (cf. Table 2). In other words, the critical findings (the CSE, as well as its relation to response repetition) appeared stable across tasks. When we calculated “conflict adaptation effects” (see “Results” section) for each task separately, we found scores of 29, 43, and 27 ms for Flanker, Simon, and Stroop tasks, respectively. This pattern disagrees with Schmidt’s (2019) recent suggestion (p. 761) that CSEs might only appear in certain tasks (e.g., the prime-probe arrow task featured in Schmidt and Weissman, 2016) but not, or only to a small extent, in other tasks such as Stroop and Flanker.

In our study, the three tasks (Flanker, Simon, Stroop) were presented in fixed blocks of rotated order. Hence, it is impossible to know at present whether the purported conflict effect in our initiation times would generalise from one task to the other. Funes et al. (2010) reported a study in which they alternated a Simon with a spatial Stroop manipulation. They found that the CSE did not generalise from one task to the other and concluded that conflict adaptation is highly specific to a given context and task. Our current results do not speak to the issue of whether conflict adaptation is general or specific. A possible follow-up experiment would be for instance to interleave Flanker with the spatial Stroop task and see whether conflict effects in initiation times are task-specific or task-general.

In summary, we used three “conflict tasks” (Flanker, Simon, Spatial Stroop) in conjunction with “mouse tracking” and focussed our interest on response movement initiation times. These appeared sensitive to the presence or absence of a conflict on the previous trial, even when initiation time on trial *N*–1 was taken into account. These results indicate a specific sensitivity to the presence or absence of recent “conflict” in the cognitive environment. This pattern, although not directly reflecting “conflict adaptation” (up- or downregulation of cognitive control in response to the presence or absence of a just-perceived conflict) in the sense originally proposed by Botvinick et al. (2001), is nonetheless clearly compatible with the general theoretical notion.

## Supplemental Material

sj-docx-1-qjp-10.1177_17470218221078265 – Supplemental material for Effects of conflict in cognitive control: Evidence from mouse tracking Click here for additional data file. Supplemental material, sj-docx-1-qjp-10.1177_17470218221078265 for Effects of conflict in cognitive control: Evidence from mouse tracking by Wenting Ye and Markus F Damian in Quarterly Journal of Experimental Psychology

## Notes

1 In mouse tracking experiments, the sampling frequency corresponds to the sampling rate of USB devices (in our case, 60 Hz, which corresponds to a sample taken every 16 ms). By contrast, reach tracking setups often have a higher sampling frequency (e.g., the equipment used in Erb et al., 2016, sampled with a frequency of 160 Hz, i.e., a sample every 6.3 ms). However, this resolution only applies to individual data points; when averaged across hundreds or even thousands of raw data points, both methods achieve millisecond resolution but with mouse tracking estimates associated with slightly higher variability than reach estimates. Hence, the difference in sampling rate is unlikely to account for the discrepancy in movement initiation times between mouse tracking and reaching.

2 We did not conduct an a priori power analysis for our study because exploration of initiation times, for which effects are bound to be small, is novel and we found it difficult to make precise predictions; hence, our study is exploratory in nature. Instead, we recruited a sample which is somewhat larger than those found in typical studies conducted in this research domain (e.g., Erb et al., 2016; Mayr et al., 2003; Wirth et al., 2020).

3 Responses in the Flanker and the Spatial Stroop task have a natural left-right orientation, but in the Simon task response key assignment is arbitrary. In our Simon task, we used the same mapping for all participants; it is acknowledged that it would be good practice to counterbalance response key assignment in future studies.

4 Note that initiation times, in addition to exhibiting highly significant task and congruency *N*–1 effects, also showed a trend towards “response repeat” emerging a potentially relevant variable. “Response repeat” codes for whether on trial *N*–1 and *N*, responses go in the same or opposite directions. If this variable, rather than being afflicted by Type I error, turns out to be relevant, this would potentially compromise our claim, based on the null finding regarding congruency on trial *N*, that participants were not yet sure of their decisions when they initiated a response movement. We find it difficult to envision a scenario which could reconcile a null finding for congruency on trial *N*, combined with a positive impact of response repetition.

5 A linear mixed-effects analysis based on a “model comparison” approach via Likelihood Ratio Tests yielded statistically equivalent results: the variables task and initiation time on trial *N*–1 made independent contributions, χ^2^ = 1,180.7 and 1,450.4, respectively, with an interactive term also improving the fit, χ^2^ = 61.9. Inclusion of the term conflict on trial *N*–1 resulted in a further improved fit, χ^2^ = 33.11, all *p*s < .001. However, inclusion of conflict on trial *N*–1 as an interactive term did not further improve the fit, χ^2^ = 5.0, *p* = .082.
