# Supplementary material for: Effects of conflict in cognitive control: Evidence from mouse tracking
Source: Q J Exp Psychol (Hove). 2022 Feb 21;76(1):54–69. doi: 10.1177/17470218221078265 (PMC9773156; doi:10.1177/17470218221078265)
Supplement: sj-docx-1-qjp-10.1177_17470218221078265 – Supplemental material for Effects of conflict in cognitive control: Evidence from mouse tracking [file sj-docx-1-qjp-10.1177_17470218221078265.docx]

Supplementary Material for:

**Effects of Conflict in Cognitive Control: Evidence from Mouse Tracking**

Wenting Ye and Markus F. Damian

**Supplementary Material A**

**Descriptives for each task (Flanker, Simon, Stroop) by condition (congruency N-1, congruency, response repeat). Standard deviations in parentheses.**

**Flanker task**

| Congruency N-1 | Congruency | Response repeat | Initiation time (in ms) | Response latency (in ms) | Maximum Deviation (in cm) |
| --- | --- | --- | --- | --- | --- |
| congruent | congruent | different | 238 (108) | 918 (195) | 6.5 (9.4) |
| neutral | congruent | different | 238 (105) | 923 (198) | 5.8 (9.0) |
| incongruent | congruent | different | 247 (109) | 930 (182) | 5.0 (7.7) |
| congruent | neutral | different | 245 (112) | 931 (190) | 7.5 (10.1) |
| neutral | neutral | different | 242 (106) | 933 (194) | 7.5 (10.3) |
| incongruent | neutral | different | 247 (117) | 928 (211) | 6.3 (8.8) |
| congruent | incongruent | different | 240 (116) | 1036 (210) | 15.5 (12.2) |
| neutral | incongruent | different | 237 (110) | 1053 (227) | 15.3 (12.2) |
| incongruent | incongruent | different | 239 (108) | 1022 (210) | 13.9 (12.1) |
| congruent | congruent | same | 237 (109) | 917 (192) | 6.1 (9.7) |
| neutral | congruent | same | 227 (106) | 918 (183) | 6.4 (9.4) |
| incongruent | congruent | same | 252 (113) | 922 (167) | 6.0 (9.3) |
| congruent | neutral | same | 232 (112) | 911 (174) | 7.9 (9.8) |
| neutral | neutral | same | 236 (109) | 916 (168) | 7.1 (10.2) |
| incongruent | neutral | same | 242 (114) | 954 (208) | 8.3 (10.5) |
| congruent | incongruent | same | 239 (112) | 1085 (227) | 17.2 (12.1) |
| neutral | incongruent | same | 231 (105) | 1044 (222) | 17.4 (11.5) |
| incongruent | incongruent | same | 239 (112) | 1031 (192) | 15.4 (11.7) |

**Supplementary Material A continued**

**Simon task**

| Congruency N-1 | Congruency | Response repeat | Initiation time (in ms) | Response latency (in ms) | Maximum Deviation (in cm) |
| --- | --- | --- | --- | --- | --- |
| congruent | congruent | different | 195 (93) | 807 (176) | 4.1 (8.7) |
| neutral | congruent | different | 184 (88) | 802 (189) | 4.6 (9.1) |
| incongruent | congruent | different | 196 (94) | 830 (198) | 5.3 (9.5) |
| congruent | neutral | different | 184 (94) | 826 (186) | 8.1 (8.9) |
| neutral | neutral | different | 190 (93) | 845 (183) | 9.7 (10.1) |
| incongruent | neutral | different | 194 (99) | 886 (202) | 11.2 (11.1) |
| congruent | incongruent | different | 190 (97) | 895 (183) | 17 (11.4) |
| neutral | incongruent | different | 190 (96) | 914 (190) | 17.4 (11.3) |
| incongruent | incongruent | different | 194 (92) | 886 (192) | 16.0 (11.2) |
| congruent | congruent | same | 189 (88) | 805 (193) | 3.3 (8.0) |
| neutral | congruent | same | 187 (87) | 786 (178) | 2.3 (7.9) |
| incongruent | congruent | same | 187 (87) | 810 (201) | 3.2 (8.2) |
| congruent | neutral | same | 193 (91) | 833 (201) | 8.6 (9.7) |
| neutral | neutral | same | 190 (91) | 815 (195) | 7.0 (9.5) |
| incongruent | neutral | same | 196 (98) | 843 (216) | 6.5 (10.1) |
| congruent | incongruent | same | 184 (86) | 924 (194) | 17.5 (10.9) |
| neutral | incongruent | same | 192 (94) | 926 (208) | 16.6 (11.3) |
| incongruent | incongruent | same | 186 (98) | 875 (210) | 13.5 (11.2) |

**Supplementary Material A continued**

**Spatial Stroop task**

| Congruency N-1 | Congruency | Response repeat | Initiation time (in ms) | Response latency (in ms) | Maximum Deviation (in cm) |
| --- | --- | --- | --- | --- | --- |
| congruent | congruent | different | 178 (82) | 814 (199) | 3.5 (8.5) |
| neutral | congruent | different | 172 (78) | 792 (187) | 3.1 (8.0) |
| incongruent | congruent | different | 173 (81) | 790 (174) | 3.8 (8.4) |
| congruent | neutral | different | 174 (85) | 812 (175) | 5.6 (8.6) |
| neutral | neutral | different | 176 (85) | 818 (178) | 7.1 (8.8) |
| incongruent | neutral | different | 181 (91) | 821 (191) | 8.3 (8.5) |
| congruent | incongruent | different | 174 (80) | 887 (183) | 16.6 (10.7) |
| neutral | incongruent | different | 177 (87) | 877 (163) | 17.4 (9.8) |
| incongruent | incongruent | different | 179 (86) | 883 (173) | 16.9 (10.6) |
| congruent | congruent | same | 173 (81) | 785 (183) | 3.6 (8.2) |
| neutral | congruent | same | 179 (81) | 781 (177) | 2.5 (7.4) |
| incongruent | congruent | same | 181 (86) | 800 (178) | 3.6 (8.5) |
| congruent | neutral | same | 178 (85) | 813 (170) | 9.2 (9.7) |
| neutral | neutral | same | 177 (87) | 762 (159) | 5.8 (8.2) |
| incongruent | neutral | same | 181 (89) | 792 (173) | 5.2 (8.4) |
| congruent | incongruent | same | 168 (84) | 925 (173) | 21.2 (10.1) |
| neutral | incongruent | same | 175 (87) | 915 (202) | 18.4 (11.0) |
| incongruent | incongruent | same | 180 (86) | 866 (176) | 14.7 (10.7) |

**Supplementary Material B**


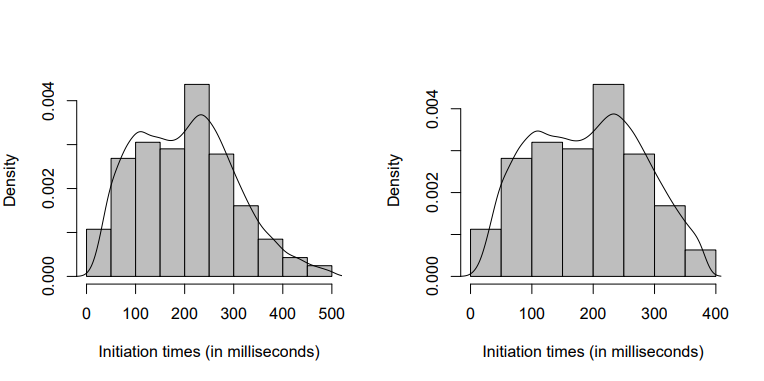


*Figure 4.* Histogram of movement initiation times. Left panel: initiation times ≤ 500 ms; right panel: initiation times ≤ 375 ms (see text for explanation).
